# Palliative care and neurology: a path to neuropalliativism

**DOI:** 10.1590/0004-282X-ANP-2022-S119

**Published:** 2022-08-12

**Authors:** Mariana Ribeiro Marcondes da Silveira, Daniel Neves Forte

**Affiliations:** 1 Universidade de São Paulo, Faculdade de Medicina, Hospital das Clínicas, Divisão de Neurologia, São Paulo SP, Brazil.; 2 Universidade de São Paulo, Faculdade de Medicina, Hospital das Clínicas, Instituto da Criança, Unidade de Dor e Cuidados Paliativos, São Paulo SP, Brazil.; 3 Universidade de São Paulo, Faculdade de Medicina, Hospital das Clínicas, Departamento de Emergências Clíncias, São Paulo SP, Brazil.; 4 Hospital Sírio-Libanês, Núcleo de Cuidados Paliativos, São Paulo SP, Brazil.

**Keywords:** Neurology, Palliative Care, Neurologia, Cuidados Paliativos

## Abstract

This article aims to expand the understanding of how it is possible to alleviate suffering and enable a dignified life trajectory for patients with progressive neurological diseases or with severe and permanent neurological impairment. The four most common disease trajectories described for people with chronic and progressive disease used to advance care planning, Brazilian normative ethical resolutions, evidence-based benefits of palliative care (PC), as well as particularities of PC in neurology, such as neurological symptom control, caring for existential and psychological suffering, care provider’s needs and particularities of pediatric neurologic PC are reviewed.

## RESUMO

Este artigo visa ampliar a compreensão de como é possível aliviar o sofrimento e possibilitar uma trajetória de vida digna para pacientes com doenças neurológicas progressivas ou com comprometimento neurológico grave e permanente. As quatro trajetórias de doença mais comuns descritas para pessoas com doença crônica e progressiva utilizadas no planejamento antecipado do cuidado, resoluções éticas normativas brasileiras, benefícios baseados em evidências dos cuidados paliativos (CP), além de particularidades dos CP em neurologia, como controle de sintomas neurológicos, cuidados existenciais e sofrimento psicológico, necessidades do cuidador e particularidades do CP neurológico pediátrico são revistos.

## INTRODUCTION

"While countless researchers work to find a cure for devastating neurological diseases, patients suffering from these diseases suffer day after day^1^". Anyone working in the field of neurology has certainly had the opportunity to follow a case of a patient with conditions as serious as a relapse(GBM), an advanced dementia syndrome or amyotrophic lateral sclerosis (ALS) in the final stage of the disease, hospitalization in an intensive care unit (ICU), with multiple complications of pneumonia, for example, and who ends up dying, inside the ICU, after numerous invasive procedures, alongside infusion pumps, vital signs monitoring devices, mechanical ventilation and dialysis , surrounded by wires, catheters and plasters.

For many years, after the completion of cardiopulmonary resuscitation attempts for patients like these, doctors would leave the room, inform the family that they had done 'everything possible', while professionals from the multidisciplinary team turned off and removed the devices, and referred the patient to the morgue. Few reflected on the following question: what is the point of taking a patient to the ICU and performing all these measures, when it is not expected that the patient can be returned to a dignified life condition?

This article aims to expand the understanding of how it is possible to alleviate suffering and enable a dignified life trajectory for patients with progressive neurological diseases or with severe and permanent neurological impairment.

## THE DISEASE TRAJECTORY

Initially, three most common disease trajectories were described for people with chronic and progressive diseases, which would be:

- Initial stability with a slow and progressive decline (a few years), followed by a more accentuated and rapid decline (a few months), a characteristic pattern of most oncological diseases and Amyotrophic Lateral Sclerosis;
- Gradual decline (a few years, average of 2-5 years), punctuated by episodes of acute deterioration and some recovery, with a more sudden and apparently unexpected death, a characteristic pattern of most respiratory, chronic heart diseases and multiple sclerosis;
- Prolonged and gradual decline (a few years, average 6-8 years), usually in an individual with some initial reduction in physical and/or cognitive reserve, with progressive accumulation of deficits that culminate in death from incidents such as pneumonia, characteristic of dementia and Parkinson's disease.

More recently, a fourth trajectory has been described, that is quite relevant for neurologists:

- Abrupt and accentuated initial loss of functionality that can result in early death, or a more uncertain prognosis, where there may be some degree of recovery and stability, or an evolution with prolonged and gradual decline, characteristic of neurological conditions with acute brain injury and severe, such as stroke, post-anoxic encephalopathy, infectious and non-infectious inflammatory conditions and traumatic brain injury (TBI)^2^ ^,^ ^3^.

Knowing which of the four trajectories best fits the patient's condition and identifying at which point he is, helps the assistant team to plan care, which includes all dimensions (physical, spiritual, psychological and social) of patient care and their caregivers, in order to alleviate suffering, and avoid situations like the one described in the introduction^4^.

## THE PATH OF ORTHOTHANASIA AND THE COST OF DYSTHANASIA

Resolution 1.805/06 of the Federal Council of Medicine (CFM), says that “in the terminal phase of serious and incurable diseases, the doctor is allowed to limit or suspend procedures and treatments that prolong the patient's life, guaranteeing him the necessary care to alleviate the symptoms that lead to suffering, from the perspective of comprehensive care, respecting the will of the patient or his legal representative”^5^. It is a resolution on orthothanasia, a word that comes from the Greek expressions orthos, (correct), and thanatos, (death). In practice, it deals with the conduct of doctors when allowing the patient to die comfortably, when his clinical state is irreversible and his death is certain^6^.

Orthothanasia differs from what was described in the introduction, which we call dysthanasia - from the Greek dis (evil) and thanatos (death). of life considered dignified by the patient, while causing suffering to the patient and his family^6^ ^,^ ^7^ ^,^ ^8^. 

The resolution of CFM 1995/2012, on the other hand, provides for the possibility that the patient can define his advance directives of will that previously express the care and treatments that the patient wants (or not) to receive at the moment when he is unable to express himself, as well as how to previously define a legal representative to express one’s values ​​and desires at this time. These directives, according to the CFM, prevail over the wishes of family members and, in order to be respected, they must not be in breach of the Brazilian medical code of ethics^9^. This is particularly important for patients with neurological conditions, who will often be unable to express themselves autonomously in later stages of the disease.

Studies also show that clinicians often report feeling that they have offered potentially inappropriate or futile care in the last six months of their patients' lives, which has been shown to be statistically significantly associated with avoidant behaviors and burn-out syndrome in these physicians. The main reason reported by them for these potentially inappropriate or futile procedures was the desire of the family^10^.

Considering that orthothanasia is foreseen by the Brazilian CFM as a practice of best interest to the patient and that dysthanasia is associated with the suffering of the patient and the assistant team plus overload of the system that finances health, how can we move towards it being allowed in the neurological patients? And what else is it possible to do for these patients, besides allowing them a dignified death?

## THE BENEFITS OF PALLIATIVE CARE (PC) AS CURRENTLY UNDERSTOOD

Many professionals still understand palliative care as relevant only at a time when there is no longer any possibility of curative treatment^4^. However, since 2002, the World Health Organization (WHO) has changed this definition. In the 21st century, PC is defined as an approach to the prevention and relief of the suffering of adults and children who have life-threatening illnesses, seeking the early identification and unerring control of this suffering in its physical, psychological, social and spiritual dimensions. In order to be achieved, they must be applied early and integrated into treatments that modify the disease, from the diagnosis of a serious disease. The objective is not to hasten death, nor to limit treatments, but to provide comfort and align the treatment of the disease with what is important for the patient^11^.

In order for these objectives to be achieved, the main competencies that the PC team must have include the ability to control pain and other symptoms, the ability to assess psychosocial aspects and communication skills^12^.

Physicians' perception that PC is only appropriate at the end of life and that patients will react negatively and give up hope if PC is offered undermines patients' access to this care^12^ ^-^ ^14^. Furthermore, contrary to the perception of physicians, an American study showed that 90% of the population studied knew very little about PC, and after reading the definition, more than 90% said that they would like to have PC available for themselves, their families and indeed for everyone, which shows that there is still a great need for efforts to educate the public and physicians^12^ ^,^ ^15^. 

A randomized study in non-small-cell lung cancer patients undergoing early PC versus standard cancer treatment showed that in the group that received early PC, there was a significant improvement in quality of life and mood, in addition to less aggressive treatments at the end of life^16^. This is often extrapolated by analogy to patients with primary tumors of the central nervous system (CNS). However, a recent systematic review did not find any studies that evaluated the impact of the early introduction of PC in neurooncology^17^, which highlights the lack of research in the area.

In Parkinson's Disease, a randomized trial of patients in need of palliative care compared patients receiving standard care, offered by a neurologist and a primary care physician, or treatment with a neurologist, a social worker, and a nurse using palliative care checklists, guided by a palliative care specialist, who was involved in selected cases. The result was improved quality of life, the amount of non-motor symptoms, the severity of motor symptoms, compliance with advanced directives, as well as a reduction in anxiety in the care provider^18^.

The study on Parkinson's Disease used an integrated palliative care model, which is usually a very good option for neurology, where the specificity of the neurological symptoms makes the presence of the neurologist important, even in cases where the moment in the disease trajectory demands PC exclusiveness. In view of the example, the following question remains: what is the current situation of PC in neurological patients?

## PALLIATIVE CARE IN NEUROLOGY: THE NEEDS ARE MANY

The most common progressive neurological diseases are Parkinson's Disease (PD), prevalence of 110-180/100,000, Multiple Sclerosis (MS), prevalence of 80-140/100,000, Amyotrophic Lateral Sclerosis (ALS), prevalence of 6-7/100,000, Progressive Supranuclear Palsy (PSP), prevalence of 7/100,000, Huntington’s Disease (HD), prevalence of 6/100,000 and Multiple System Atrophy (MSA), prevalence of 5/100,000^19^. In addition to these, other conditions such as primary tumors of the central nervous system (23.8/100,000)^20^, cerebrovascular accident (CVA) and extensive traumatic brain injury (TBI), as well as dementia syndromes (12.1/1000)^21^ significantly increase the prevalence of neurological patients benefiting from palliative care. Despite the progress related to these diseases in recent years, more than one billion people in the world have neurological diseases and more than one in 10 deaths are caused by neurological diseases; most neurological diseases remain incurable, reducing length and quality of life^22^.

Even with some neurological symptoms and types of trajectory common among these diseases, each one of them has very specific particularities in terms of symptoms and symptomatic treatment^19^, which makes the neurologist's contribution to the diagnosis of diseases, management of symptoms, advances in the treatments and fundamental prognosis for the establishment of effective PC^1^. The concept of PCin neurology is constantly evolving, but there is still a lot of stigma. Many neurologists persist in understanding that offering PC to neurological patients is a doctor's responsibility, because they continue to associate PC exclusively with end-of-life care and the “lack of things to do”. However, the evolution of the concept of PC, as defined above, and the efforts of neurology societies to disseminate these concepts have gradually expanded the scope of PC^23^.

In a 1996 position statement, the American Academy of Neurology (AAN) Ethics and Humanities Subcommittee declared that primary PC is the responsibility of all neurologists, and this position remains unchanged^24^. In 2008, the European PC association also created a task force with the European Federation of Neurological Societies to investigate PC in neurological diseases^19^. During the 2017 AAN meeting, a group of neurologists and palliative care specialists met to set clinical, research and educational priorities in the field that has been defined as neuropalliativism. At this meeting, the “neuropalliative care approach” was defined as care focused on the specific needs of patients with neurological diseases and their families, including primary palliative care (provided by the patient's primary care team including the neurologist) and specialized palliative care (provided by clinicians with subspecialty in palliative care)^22^. Thus, neuropalliative care represents an emerging subspecialty within neurology and palliative care and a new way of approaching people with neurological disorders^24^.

Faced with all these challenges and recognizing the importance of the topic, the Brazilian Academy of Neurology created in 2020 a Palliative Medicine Center^25^. In 2022, the AAN published a new guideline, already using the concept of neuropalliative care (NC)^24^.

During the 2017 AAN meeting that brought together experts from around the world to debate neuropalliative care, a group of PC skills that every neurologist should have was defined: (a) Identify common palliative care needs associated with specific neurologic disorders; (b) Detect and manage whole body pain; (c) Provide basic psychosocial and spiritual support; (d) Acquire communication skills including empathetic listening; (e) Effectively estimate and communicate prognosis and uncertainty; (f) Master shared decision-making for common preference sensitive decisions; (g) Master shared decision-making and support for patients and families around tragic choices; (h) Be aware of palliative care options of last resort; (i) Recognize and manage caregiver distress and needs^22^.

In addition, priorities were defined for the development of NC in clinical, educational and research aspects.

From a clinical point of view, there are no evidence-based models showing what would be the best strategy to integrate PC with neurological care, with primary palliative care and specialized palliative care as an option, in consultative models, where traditional neurological treatment is performed. by the neurologist while the PC specialist works separately, or in a simultaneous model, where PC is divided between neurologists and palliative care specialists. In addition, a priority to create and implement quality measures to assess end-of-life care, symptom screening and documentation of values ​​and objective of care was raised.

From an educational point of view, numerous objectives were established, such as reducing the stigma of PC among physicians, patients and family members, establishing a grid of content and skills that all residents should develop, encouraging the creation of opportunities for learning neuropalliative care, including undergraduate teaching, residency and dissemination through workshops, among others.

From a research point of view, there exists a search for a better understanding of the natural history of neurological diseases, the improvement of prognostic tools, symptomatic treatments, understanding of the way people make decisions and the best way to integrate PC into neurological practice^22^.

As such, there is a growing commitment to the duty of all neurologists to know and practise the principles of PC and to recognize when more complex cases will benefit from a team specializing in PC^24^.

A study with eight centers in the United Kingdom to assess the integration of neurology and PC services published in 2016, showed heterogeneity not only between the different centers evaluated, but also between different neurological diseases. There was a better integration between the two specialties in cases of motor neuron diseases, followed by cases of parkinsonism (including PD, MSA and PSP), these being lower in cases of BD^26^.

Another study carried out with neurology and palliative care specialists from six centers in the United Kingdom, seeking to understand how professionals from both groups see the integration of the two areas, showed, in responses to questionnaires sent by email, that 58% of PC specialists and only 36% of neurologists rated the relationship between specialties as good or excellent. Even with a low response rate (20% of the specialists responded to the questionnaires), the nature of the responses suggests that there is much room for increased collaboration between the two specialties^27^.

To make more evident the differences in the model of understanding of neurological diseases that may prevent good collaboration between palliative care specialists and neurologists, Table 1, taken from Glover TL and Kluger BM^28^ summarizes the palliative care model. and the more common model, where neurological diseases are seen exclusively as chronic diseases.

**Table 1.** Palliative care model vs chronic illness model.

Palliative care	Chronic illness
Primary goal is to relieve suffering	Primary goal is to preserve function
Care is provided to patient, caregiver, and other family members	Care is centered around patient-physician dyad
Accepts death and decline as expected outcomes and plans accordingly	Death is viewed as an adverse outcome and focus is on prolonging life
Addresses psychosocial and spiritual issues in addition to medical symptoms	Focus is on medical and psychiatric symptoms
Team approach is essential	Variable use of team approach

In order for integration to take place, neurology societies have organized themselves, as we explore in this article. Recognizing the need for palliative care in neurology, including creating the concept of neuropalliative care, is to understand that PC in neurological diseases has its particularities^29^, and this will be the next topic.

## PARTICULARITIES OF NEUROLOGICAL CARE

### Neurological symptoms

In general, regardless of the etiological diagnosis, patients with more severe neurological impairment can be expected to have greater functional impairment, a greater number of neurological and clinical symptoms and a more reserved prognosis. Knowing the severity and topography of the neurological involvement makes it possible to anticipate what symptoms and clinical complications to expect^30^ ^,^ ^31^. 

CNS cancer, for example, usually differs from other neoplasms by the higher incidence of seizures, cognitive decline, headache and focal neurological deficits; control of delirium and agitation in a patient with Alzheimer's Disease or Lewy Body Dementia requires separate management of delirium in a clinical setting in a patient without underlying dementia^29^; The possibilities for managing mechanical respiratory failure vary in the different types of neuromuscular diseases and are different from patients with respiratory failure due to conditions such as Chronic Obstructive Pulmonary Disease.

If the patient's desire is to prolong life as much as possible, in a condition such as Duchenne Muscular Dystrophy, tracheostomy will be necessary if the patient cannot cooperate with non-invasive ventilation and use of mechanical insufflation-exsufflation methods, such as in acute conditions that increase secretions too much and lower the level of consciousness. In a patient with motor neuron disease such as ALS with bulbar involvement, the vocal cord spasticity typical of this condition will reduce the insufflation-exsufflation flow to values ​​that prevent the maintenance of non-invasive ventilation in an advanced stage of the disease^32^ ^,^ ^33^. 

In addition, the loss of mobility, communication skills and cognitive function, often long before death, can make access to outpatient follow-up difficult, hindering the identification and treatment of controllable clinical symptoms. Furthermore, timely conversations about advance directives for end-of-life treatments may be lost^1^.

### Existential and psychological suffering

An often prolonged and fluctuating course of neurological diseases, with unexpected declines and gradual accumulation of deficits, can lead to increased needs to support the distress of sequential losses^1^, which many family members report as the impression of losing a loved one still in life^29^.

Acute brain injuries can abruptly and very significantly reduce the level of consciousness of these patients, which can leave the family without knowing how to act with a person who has completely changed.

In addition, unlike cancer, which is considered something extrinsic to the person and against which one can fight, neurological diseases are seen in a more juxtaposed way to the person, in such a way that the disease is often confused with the patient. This can lead to a situation in which the impacts of neurological diseases on the person's functionality, such as memory and attention deficits, inappropriate behaviors and lack of coordination, are seen as personal failures^29^.

### Caregiver's needs

The pattern of neurological symptoms, with a high impact on patients' functionality, associated with often prolonged disease trajectories, increase the burden on the caregiver. In addition, the need to represent the patient's values ​​when he is unable to express his wishes autonomously occurs more frequently when compared to other diseases. Initial cognitive decline increases patients' feelings of worthlessness, with a greater risk of depression in patients and their caregivers.

Furthermore, the lack of reliable prognostic parameters in neurological diseases is commonly associated with uncertainties about the prognosis and disease trajectory, which causes considerable distress and can make treatment decisions and advance directives difficult^29^ ^,^ ^31^. 

### Particularities of pediatric neurology

The family may feel guilty believing that they did something inappropriate in the perinatal period, or that they passed on the gene responsible for the disease in neurogenetic syndromes, particularly when more than one child is affected. Guilt over past issues can share space with intense concerns about future issues, such as how a child who will never have autonomy will survive financially if he reaches adulthood.

In addition, family members of children who do not communicate may have to work hard to make them believe in the needs they identify in their children.

Causes of grief can have some peculiarities that include suffering from the need to justify to other people all the time the love they feel for their children who are different from other children. When children die, there is also a need to reframe a life previously dedicated to caring for the child.

In addition, fears about the child's future, including the challenges of transitioning care from pediatric to adult services, accompany these family members^31^.

Thus, the competence in neurology and in the control of non-neurological symptoms common to neurological patients is added to the need to develop communication techniques and expand the understanding of grief, in order to be able to contemplate all the complex demands of these patients.

### Communication and grief

When humans face situations considered dangerous, such as the abrupt awareness of the possibility of death, it is common for them to adopt a pattern of acute response to stressors such as “flight, freeze or fight”. So it's not uncommon for a patient to report "not hearing anything else" after the doctor says "you have Alzheimer's disease." The automatic response overrides cognitive functions. Thus, when giving difficult news, one of the most important skills is to recognize the patient's emotions and respond to them, leaving information about the diagnosis and conduct for a moment when the patient can take it in^34^.

Considering an example where the patient expresses that “the hallucinations and vivid dreams I have are making me terrified”, we can use the technique based on the acronym NURSE, described in Table 2, adapted from Back et al.^34^.

**Table 2.** responding to emotions with words, adapted from Back et al.^34^.

N	NAME the emotion	“I can see that you are frightened about...”
U	UNDERSTAND the emotion	“It must be so hard to be in a situation like that”
R	RESPECT the patient	“I’m so impressed that you’ve been able to keep up with your daily life while having these hallucinations during the day and disturbing dreams at night.”
S	SUPPORT the patient	“I will be here, and my team, to help you with these symptoms.”
E	EXPLORE the emotion	“Tell me more about how these hallucinations and vivid dreams are affecting you.”

Approaches of this kind are more effective than common-sense words like “I know what you are feeling,” when the truth is that even if the doctor had already experienced the same symptoms, the fact that he is a totally different person from the patient already makes it impossible to truly know what he feels^35^.

In addition to responding to emotions with words, it is important that non-verbal communication is consistent with words. One way to become more aware of your body is to remember the acronym S-O-L-E-R, where: (S) Face the patient *squarely* to indicate interest; (O) adopt an *open* body posture; (L) *lean* toward the patient; (E) use *eye* contact to show you are paying attention; and (R) maintain a *relaxed* body posture^34^.

To systematize the most important steps for communication, the mental map described by the acronym SPIKES can be quite useful, as described in Table 3, adapted from Back et al.^34^.

**Table 3.** A cognitive map for talking about serious news, adapted from Back et al.^34^.

S	Setup	Prepare the ambient (information you need, quiet place to sit down, water and box of tissues...)
P	Perception	Assess the patient’s perception: “What have other doctors told you so far?”
I	Invitation	Ask for an invitation to talk about the news: “Could we talk about the news?”
K	Knowledge	Disclose the news straightforwardly: “The neuroimaging came back, and there is some serious news that we need to discuss.”
E	Emotion	Respond to the patient’s emotion: recognize and respond, according to previously mentioned.
S	Summarize	Summarize the plan: summarize what you’ve discussed and the next steps the patient will need to take.

What follows the information about the disease, even if given in an empathic and respectful way, was described in several models, such as Grief work, originating from Freud's work in "Mourning and Melancholia", Attachment Theory described by Bowlby, the phases of acceptance by Kübler-Ross and the dual process of coping with bereavement by Stroebe and Schut. A more detailed review of these models is beyond the scope of this review. However, it is worth reflecting a little more on the dual process of coping with bereavement, as it reflects one of the most contemporary ways of looking at the process of coping with bereavement, which involves: (a) orientation towards loss, (b) orientation towards the reset and (c) oscillation.

When the orientation is towards loss, the bereaved individual's attention is focused on aspects of illness or death, which can be exemplified by the individual who has just received a serious diagnosis and is deeply distressed by the loss of functionality and cognitive capacity that will come.

At the moment of guidance for reestablishment, there are secondary consequences to the loss that constitute sources of stress with which the bereaved person needs to deal, as well as the definition of ways of doing it, which can be exemplified by the bereaved person thinking about taking advantage of the best lucid and functional time he will have in the presence of his children and grandchildren, for instance organizing Sunday lunches.

The oscillation between the orientation towards loss and the orientation towards restoration appears as the most distinctive dimension of this model when compared to the previous ones^36^ ^,^ ^37^. 

Recognizing the moments of guidance for loss and for reestablishment, and that the bereaved individual can oscillate between them, often during the same conversation, is essential for a process of reception and support for effective psychic suffering. Studying and training to recognize and respond to the patient's emotions using appropriate verbal and non-verbal language, and to enable the difficult conversations that are intrinsic to the trajectory of neurological diseases in a technical and empathic way, allows medical care to be offered in an assertive way.

## NOT TO FINISH...

For palliative care in general, and neuropalliative care specifically, to be able to happen, either as a palliative intention or in a specialized context, these must first be known as a possibility. We hope to have fulfilled the objective of presenting them here in general terms.

In an interview by Dr Stacey Clardy with behavioral neurologist Dr Daniel Drubach about his experience of being diagnosed with Lewy Body Dementia, he talks about the possibility of rebuilding the diseased brain. It is not an experimental treatment, but an attempt by the bereaved individual, faced with a neurodegenerative disease, to rebuild himself in the present brain and body, even with limitations, suffering and uncertainties^38^.

Both in the interview given to the Neurology Podcast^38^ and in the article published in a recent issue of Continuum^35^, Dr. Daniel seeks to explore the symptoms that appear and how his body behaves in the face of them, while reviewing moments in his biography that correspond with the symptoms.

Dr. Daniel seeks to give meaning to his life in the present dimension. This is possible due to the nature of personality and choices, but it is certainly supported by a support network of family members and health professionals. Those who practise palliative and neuropalliative care, specialized or primary, must offer a technical and empathic approach, so that the patient and their surroundings have the possibility to live the experience of the disease and the grieving process according to their values, in a dignified way and with controlled suffering.

Only then will situations of therapeutic obstinacy and dysthanasia as described in the introduction become things of the distant past.
